# No association between *Helicobacter pylori* infection and diabetes mellitus among a general Japanese population: a cross-sectional study

**DOI:** 10.1186/s40064-015-1371-2

**Published:** 2015-10-13

**Authors:** Takashi Tamura, Emi Morita, Sayo Kawai, Tae Sasakabe, Yuka Sugimoto, Nana Fukuda, Shino Suma, Hiroko Nakagawa, Rieko Okada, Asahi Hishida, Mariko Naito, Nobuyuki Hamajima, Kenji Wakai

**Affiliations:** Department of Preventive Medicine, Nagoya University Graduate School of Medicine, 65 Tsurumai-cho, Showa-ku, Nagoya, 466-8550 Japan; Department of Epidemiology and Preventive Medicine, Gifu University Graduate School of Medicine, 1-1 Yanagido, Gifu, 501-1194 Japan; Environmental Planning Laboratory, Department of Forest Management, Forestry and Forest Products Research Institute, National Research and Development Agency, 1 Matsunosato, Tsukuba, 305-8687 Japan; Department of Oral Disease Research, National Center for Geriatrics and Gerontology, 7-430 Morioka-cho, Obu, 474-8511 Japan; Division of Epidemiology and Prevention, Aichi Cancer Center Research Institute, 1-1 Kanokoden, Chikusa-ku, Nagoya, 464-8681 Japan; Department of Healthcare Administration, Nagoya University Graduate School of Medicine, 65 Tsurumai-cho, Showa-ku, Nagoya, 466-8550 Japan

**Keywords:** *Helicobacter pylori*, East Asian CagA, Diabetes mellitus, Cross-sectional study, Japan

## Abstract

Several case-control studies have reported that patients with diabetes mellitus (DM) had a higher prevalence of *Helicobacter pylori* infection than those without DM, but these findings remain equivocal. Additionally, there are few studies examining associations between East Asian CagA-positive *H. pylori* and DM. This cross-sectional study aimed to investigate whether *H. pylori* infection was a possible risk factor for DM in a general Japanese population. The study included 5165 subjects (1467 men, 3698 women) aged 35–69 years from the Daiko Study, part of the Japan Multi-Institutional Collaborative Cohort Study. A urinary anti-*H. pylori* antibody was used to detect *H. pylori* infection. The medical history of physician-diagnosed DM was confirmed by self-administered questionnaire. The odds ratios (ORs) and their 95 % confidence intervals (CIs) for DM (current and former) were calculated using unconditional logistic regression analysis, adjusting for age, sex, educational status, alcohol use, smoking status, body mass index, energy intake, and physical activity. The prevalence of DM was 4.6 % (95 % CI 3.7–5.6 %) among 1878 participants with *H. pylori* infection and 3.2 % (2.6–3.8 %) among 3287 without the infection (*p* = 0.009). The crude, age-adjusted, and multivariate-adjusted ORs for DM in those with the infection relative to those without were 1.47 (95 % CI 1.10–1.97), 1.02 (0.76–1.38), and 0.97 (0.71–1.32), respectively. We found a significantly higher DM prevalence among those with *H. pylori* infection than among those without. However, almost all the difference in prevalence could be explained by the older age of those infected. Our findings did not support an association between *H. pylori* infection and DM.

## Background

*Helicobacter pylori* infection is a major risk factor for gastric cancer (Montecucco and Rappuoli 2001) and is also well known as a causative factor for ulcers, atrophic gastritis, mucosa-associated lymphoid tissue lymphoma, and intestinal metaplasia (Labenz and Borsch 1994; Asaka et al. 2001; Wundisch et al. 2005). *Helicobacter pylori* infection has been estimated to be present in half of the world’s population, and it has been reported to be higher in developing countries than in industrialized countries (Suerbaum and Michetti 2002; Correa and Piazuelo 2008).

Some studies reported that *H. pylori* is a possible risk factor for cardiovascular disease and metabolic syndrome, potentially mediated by elevations in inflammatory molecules, such as C-reactive protein and interleukin-6 (Georges et al. 2003; Nabipour et al. 2006). *Helicobacter pylori* infection has been linked with the activation of toll-like receptors, resulting in energy harvesting and fat accumulation, which lead to insulin resistance (Manco et al. 2010). Several cross-sectional studies showed that *H. pylori* infection was associated with diabetes mellitus (DM) (Chen and Blaser 2012; Hsieh et al. 2013; Han et al. 2015). A recent prospective cohort study also reported that *H. pylori* infection was correlated with a significantly high hazard ratio for DM (Jeon et al. 2012). However, the association between *H. pylori* infection and the risk of DM remains controversial, because several cross-sectional studies reported that *H. pylori* was not associated with insulin resistance or the prevalence of DM (Ko et al. 2001; Anastasios et al. 2002; Howard et al. 2008; Lutsey et al. 2009), and there are few studies reporting such associations among large populations in East Asia, where the more pathogenic East Asian CagA-positive strain of *H. pylori* is dominant (Fock and Ang 2010).

We previously reported the prevalence of *H. pylori* infection in 2008–2010 among a large general Japanese population (Tamura et al. 2012). In the present study, we aimed to investigate whether or not *H. pylori* infection was associated with DM in a cross-sectional study in the same population.

## Methods

### Subjects, lifestyle, and medical history of diabetes mellitus

The study included 5165 participants (1467 men, 3698 women) aged 35–69 years in the Daiko Study, part of the Japan Multi-Institutional Collaborative Cohort Study (J-MICC Study) (The J-MICC Study Groups 2007; Morita et al. 2011). The participants were enrolled at the Daiko Medical Center from June 2008 to May 2010.

All participants provided written informed consent and completed a questionnaire on their lifestyle, including education, alcohol use, smoking status, exercise, dietary habits, history of *H. pylori* eradication treatment, and medical history of physician-diagnosed DM (current, former, or never). Of 5172 initial participants, we excluded four who withdrew from the study, two with no answer about their DM medical history, and one without a urine sample for the detection of anti-*H. pylori* antibody. The J-MICC Study and the Daiko Study were both approved by the Ethics Review Committee of Nagoya University School of Medicine (approved numbers were 253 and 618, respectively).

### Detection of *H. pylori* infection

An antibody kit for urine, Rapiran (Otsuka Pharmaceutical Co., Ltd., Tokyo, Japan) was used to detect *H. pylori* infection. The sensitivity and specificity of the assay were 89.6 and 93.8 %, respectively, based on biopsies of gastric mucosa as a gold standard (Yamamoto et al. 2000; Graham and Reddy 2001; Fujisawa et al. 2001).

### Estimation of physical activity levels

The daily metabolic equivalent (MET)-hours/day (MET levels × hours/day) were estimated based on our questionnaire. Subjects were asked about the average hours per day (none, <1, 1 to <3, 3 to <5, 5 to <7, 7 to <9, 9 to <11, and ≥11 h) of four types of physical activity: heavy physical work, walking, standing, and sitting. The following time scores were assigned: 0 for none, 0.5 for <1, 2 for 1 to <3, 4 for 3 to <5, 6 for 5 to <7, 8 for 7 to <9, 10 for 9 to <11, and 11 for ≥11 h. On the basis of previous studies (Inoue et al. 2008a, b), the following MET scores were assigned: 4.5 for heavy physical work, 3.0 for walking, 2.0 for standing, and 1.5 for sitting. We defined and included only ≥3.0 METs activity as physical activity, and each MET-hours/day (i.e., heavy physical work and walking) was calculated by multiplying the MET intensity for each activity by the daily time score for each activity.

Leisure-time physical activity was calculated using the international physical activity questionnaire (IPAQ) as in a previous study (Higashibata et al. 2012). IPAQ was developed and validated as an instrument for cross-national monitoring of physical activity and inactivity (Craig et al. 2003). Total MET-hours/day (total physical activity) were then estimated by adding the sum of daily physical activity to that of leisure time for those who responded to all the questions (n = 5101).

### Estimation of total energy intake

Energy intake (kcal/day) was estimated using a short food frequency questionnaire for each subject. The questionnaire has been validated for estimation of energy and nutrient intakes in Japanese subjects by referring to dietary records (Tokudome et al. 2004, 2005; Goto et al. 2006; Imaeda et al. 2007).

### Statistical analysis

Differences in mean values between those with and without *H. pylori* infection were examined by the Student *t* test, and the differences in proportions were tested using the Chi square test. Confidence intervals (CIs) of *H. pylori* infection or DM prevalence were estimated based on a binominal distribution.

We calculated a prevalence rate ratio of DM for the infection. Additionally, the following odds ratios (ORs) of DM (current or former) for *H. pylori* infection (positive) with their 95 % CIs were estimated using unconditional logistic regression models: crude OR, age-adjusted OR, age- and sex-adjusted OR, and multivariate-adjusted OR, adjusted for age (as a continuous variable), sex, education (≤12, 13–15, or ≥16 years), alcohol use (current, former, or never), smoking status (current, former, or never), body mass index (BMI, as a continuous variable), energy intake (as a continuous variable), and total physical activity (as a continuous variable) as confounders. Those who drank at least once a month were defined as current drinkers. Those with any covariate missing were excluded from the multivariate analysis (n = 85).

The statistical power to detect given ORs for DM with an α error of 0.05 was calculated based on the sample size, the infection rate of *H. pylori*, and the DM prevalence after study completion. A *p*-value of <0.05 was considered statistically significant. All the statistical analyses were performed using STATA/IC 12.1 (STATA Corp., College Station, TX, USA).

## Results

Table 1 compares the characteristics of 5165 study subjects between those with *H. pylori* infection and those without. In total, 28.4 % were men and 71.6 % were women. Mean age ± standard deviation was 53.6 ± 10.3 years in men and 52.2 ± 10.3 years in women, with 58.5 % of participants aged ≥50 years. A total of 190 participants reported a history of DM (current or former) in the questionnaire. Participants without *H. pylori* infection tended to have a higher education. The proportions of current drinkers were 55.0 % for those without infection and 52.6 % for those with infection, while respective proportions of current smokers were 11.3 and 12.7 %; these differences were not statistically significant. Means of age and BMI were significantly higher in those with *H. pylori* infection than those without (*p* < 0.001 and *p* = 0.002, respectively). Mean total physical activity was marginally higher in those with the infection than in those without (*p* = 0.09). Average energy intake was not significantly different between those with and without infection.

**Table 1:** Background characteristics of the study subjects according to *H. pylori* infection

Characteristic	Total (n = 5165)	*H. pylori*		
		Uninfected (n = 3287)	Infected (n = 1878)	*p*
Age (years)				
Mean ± SD	52.5 ± 10.3	50.7 ± 10.3	55.8 ± 9.6	<0.001
Range	35–69	35–69	35–69	
Sex, n (%)				
Men	1467 (28.4)	891 (27.1)	576 (30.7)	0.006
Women	3698 (71.6)	2396 (72.9)	1302 (69.3)	
Diabetes mellitus, n (%)				
Current	156 (3.0)	84 (2.6)	72 (3.8)	0.07
Former	34 (0.7)	20 (0.6)	14 (0.8)	
None	4975 (96.3)	3183 (96.8)	1792 (95.4)	
Education, n (%)				
≤12 years	2088 (40.4)	1213 (36.9)	875 (46.6)	<0.001
≥13–15 years	1535 (29.7)	1025 (31.2)	510 (27.2)	
≥16 years	1524 (29.5)	1037 (31.5)	487 (25.9)	
Unknown	18 (0.4)	12 (0.4)	6 (0.3)	
Alcohol use, n (%)				
Current	2798 (54.2)	1809 (55.0)	989 (52.6)	0.24
Former	105 (2.0)	61 (1.9)	44 (2.4)	
Never	2261 (43.8)	1416 (43.1)	845 (45.0)	
Unknown	1 (0.02)	1 (0.03)	0 (0.0)	
Smoking status, n (%)				
Current	609 (11.8)	371 (11.3)	238 (12.7)	0.10
Former	1025 (19.8)	636 (19.3)	389 (20.7)	
Never	3530 (68.3)	2280 (69.4)	1250 (66.6)	
Unknown	1 (0.02)	0 (0.0)	1 (0.05)	
BMI (kg/m^2^)^a^				
Mean ± SD	21.7 ± 3.2	21.6 ± 3.2	21.9 ± 3.2	0.002
Range	11.5–52.8	13.6–42.4	11.5–52.8	
Energy intake (kcal/day)				
Mean ± SD	1612 ± 326	1611 ± 324	1614 ± 329	0.35
Physical activity (MET-hours/day)				
Mean ± SD	14.5 ± 9.0	14.3 ± 8.9	14.8 ± 9.1	0.09

*BMI* body mass index, *SD* standard deviation, *MET* metabolic equivalent

^a^Data were missing for one subject with no information on body weight

**Fig. 1:**
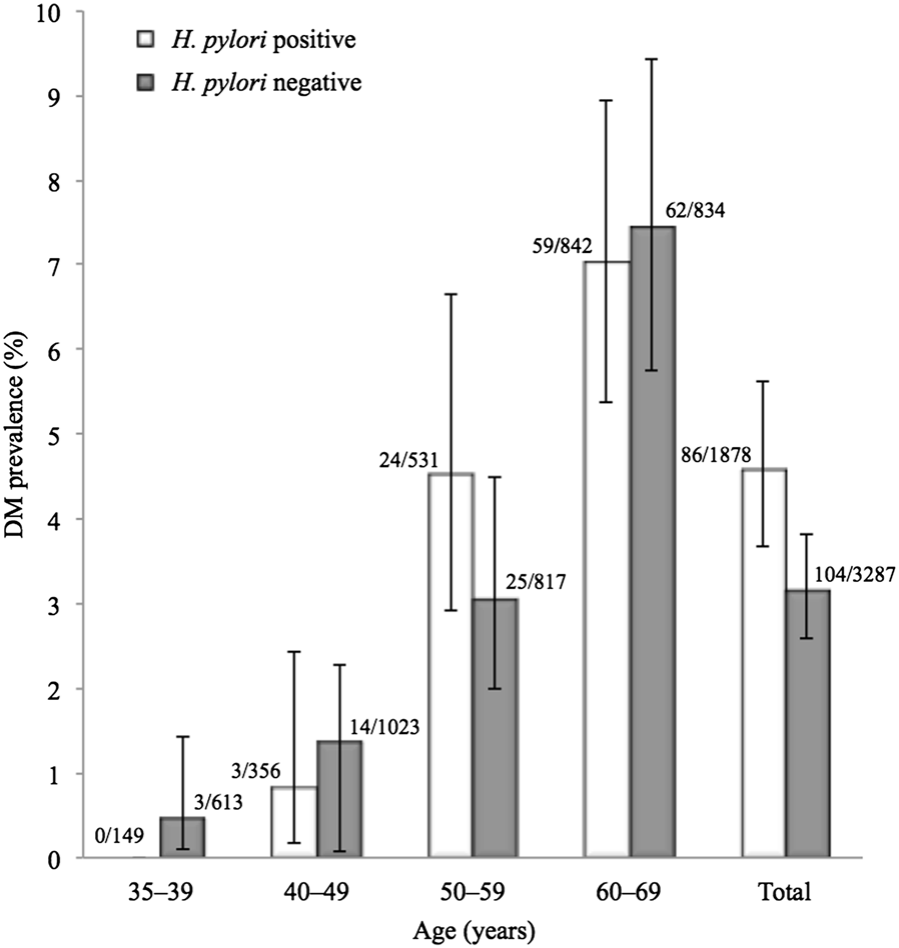
Prevalence of diabetes mellitus among those with *H. pylori* infection and those without. *DM* diabetes mellitus. *Numbers* at *tops of bars* show n of DM (+)/n. *Error bars* indicate 95 % confidence intervals

Figure 1 shows the DM prevalence according to infection status and age. The prevalence was 4.6 % (95 % CI 3.7–5.6 %) among those with *H. pylori* infection and 3.2 % (95 % CI 2.6–3.8 %) among those without, resulting in a significant difference (*p* = 0.009). The difference within each age group, however, did not reach statistical significance.

Table 2 summarizes the ORs for DM in relation to *H. pylori* infection. The prevalence rate ratio and the crude OR of DM for those with the infection relative to those without were greater than unity: 1.45 (95 % CI 1.09–1.91) and 1.47 (95 % CI 1.10–1.97), respectively. The age-adjusted OR, however, showed no association: 1.02 (95 % CI 0.76–1.38). The age- and sex-adjusted and the multivariate-adjusted ORs also indicated no association: 0.98 (95 % CI 0.72–1.32) and 0.97 (95 % CI 0.71–1.32), respectively. Including 167 *H. pylori*-negative subjects who had a history of eradication treatment as positive subjects had little effect on the ORs (date not shown).

**Table 2:** Prevalence rate ratio and odds ratios and 95 % confidence intervals for diabetes mellitus in relation to *H. pylori* infection

	*H. pylori*	
	Uninfected (n = 3287)	Infected (n = 1878)
Diabetes mellitus		
Never, n (%)	3183 (96.8)	1792 (95.4)
Current or former, n (%)	104 (3.2)	86 (4.6)
Prevalence rate ratio	1	1.45 (1.09–1.91)
Crude OR	1	1.47 (1.10–1.97)
Age-adjusted OR	1	1.02 (0.76–1.38)
Age- and sex-adjusted OR	1	0.98 (0.72–1.32)
Multivariate-adjusted OR^a^	1	0.97 (0.71–1.32)

*OR* odds ratio

^a^Adjusted for age, sex, education, alcohol use, smoking status, BMI, energy intake, and total physical activity

## Discussion

In this study, we examined the association of *H. pylori* infection with medical history of DM. Overall, DM prevalence was significantly higher in those with *H. pylori* infection than those without infection. However, after adjusting for age as a confounder, *H. pylori* infection was not associated with DM in a general Japanese population.

*Helicobacter pylori* is transmitted from person to person through oral-oral or fecal-oral routes during early childhood (from 1 to 3 years old), and the infection usually persists unless eradication therapy has been successful (Goodman and Correa 1995). Infection among adults is possible, but highly limited (Brown 2000). Thus, *H.pylori*-positive subjects in this study would have been infected for decades (Tamura et al. 2012). Several case-control studies have reported that *H. pylori* infection was significantly associated with DM (Bener et al. 2007; Devrajani et al. 2010). Additionally, a prospective cohort study and a meta-analysis of observational studies also showed that *H. pylori* infection is a risk factor for DM (Jeon et al. 2012; Zhou et al. 2013). Some biological mechanisms may explain the association. First, altered glucose metabolism may produce chemical changes in the gastric mucosa that help to detect *H. pylori* infection (de Luis et al. 1998). Second, *H. pylori* gastric infection increases secretion of proinflammatory cytokines, resulting in changes in the structure of insulin receptor interfering with the interaction between its receptor and insulin (Bener et al. 2007). Our study also found a significantly higher prevalence rate ratio and crude OR of DM in relation to *H. pylori* infection. However, the age-adjusted OR was almost unity, which means that the significant difference in prevalence of DM between those with and without the infection could be explained by the older age of those infected and the higher prevalence of DM in the elderly. This explanation was further supported by the fact that DM prevalence was not significantly different between infection statuses in each age stratum (Fig. 1). Since the risk of DM is influenced by lifestyle factors such as alcohol use, smoking status, dietary habits, and exercise, we estimated the multivariate-adjusted ORs of DM according to infection status, and again found no association. Our results indicate that *H. pylori* infection may not be a risk factor for DM in Japanese subjects. This is in accordance with other studies that found negative or neutral results for such an association (Demir et al. 2008; Ciortescu et al. 2009).

A previous meta-analysis for the association between *H. pylori* infection and DM reported a significantly high OR (1.33, 95 % CI 1.08–1.64) (Zhou et al. 2013), although the analysis mainly included case-control studies and only a few studies in East Asian populations. Our findings in a large Japanese population should contribute to understanding the association in East Asian individuals. The East Asian CagA-positive strain of *H. pylori* is a well-known risk factor for gastric cancer (Azuma et al. 2004). Interestingly, there are two major subtypes of CagA, the East Asian and the Western types, and the former subtype is dominant in Japan except for Okinawa Prefecture (Azuma 2004) on the southernmost islands of the country. Only one-half to two-thirds of Western infections carry Western CagA, while nearly all East Asian strains have East Asian CagA (Van Doorn et al. 1999). Some previous studies reporting the association between *H.pylori* and DM suggested that Western CagA increased the risk of DM (Jeon et al. 2012; Zhou et al. 2013). In contrast, our study may indicate that the East Asian CagA strain is not associated with DM. Infection with the East Asian CagA-positive strain is clearly involved in the pathogenesis of gastric cancer, with persistent inflammation induced by CagA. However, the increased inflammation may not necessarily enhance insulin resistance and fat accumulation as has been suggested in previous studies in Asian populations (Howard et al. 2008; Lutsey et al. 2009). The mechanism by which only Western strains induced DM remains to be elucidated.

Our study involved a large population that lends support to our conclusion. There are limitations in this study. First, fasting blood glucose level and glycosylated hemoglobin (HbA_1c_) values were not directly measured. However, the sensitivity and specificity of our questionnaire for DM were 74.4 and 98.1 %, respectively, when based on a HbA_1c_ (National Glycohemoglobin Standardization Program) value ≥6.5 %, which was the gold standard for diagnosis of DM among 5009 subjects in the Shizuoka area, in another part of the J-MICC study (Asai et al. 2009). Therefore, we believe that case ascertainment using self-report was reasonably valid for the present study. Second, we could not differentiate the type of DM (type 1 or 2) due to the limitation of data, but the prevalence of type 1 DM in Japan is much lower than that of type 2 DM (International Diabetes Federation 2013). Third, we did not use the well-validated serological tests for *Helicobacter pylori* infection. However, according to previous studies, the sensitivity and specificity of urinary test used in our study are similar to those of the validated serological tests (Yamamoto et al. 2000; Burucoa et al. 2013). We employed the urinary test because it could be superior to serum tests in terms of the convenience and non-invasiveness to participants. Finally, this study had statistical power of 69.2, 74.6, and 83.6 % to detect ORs of 1.45, 1.50, and 1.55, respectively, for the infected *versus* uninfected. The power was 43.7 %, if the true OR was 1.33 based on a previous meta-analysis (Zhou et al. 2013). This study had a relatively high *β* error possibility (i.e., 56.3 %) to overlook such an association, which means that a slight increase in the risk for DM in relation to *H. pylori* cannot be ruled out.

## Conclusion

Our findings suggest that infection of East Asia CagA-positive *H. pylori* is not a risk factor for DM. Further prospective cohort studies of non-DM subjects with or without *H. pylori* infection are warranted.

## Abbreviations

BMI: body mass index
CI: confidence interval
DM: diabetes mellitus
HbA_1c_: glycosylated hemoglobin
*H. pylori*: *Helicobacter pylori*
IPAQ: international physical activity questionnaire
J-MICC: Japan-Multi Institutional Collaborative Cohort
MET: metabolic equivalent
OR: odds ratio
SD: standard deviation
